# A Path to Prediction of Outcomes in Juvenile Idiopathic Inflammatory Myopathy

**DOI:** 10.3389/fimmu.2019.00638

**Published:** 2019-04-02

**Authors:** Ann Marie Reed, Cynthia S. Crowson, Jeffrey Arthur Dvergsten

**Affiliations:** ^1^School of Medicine, Duke University, Durham, NC, United States; ^2^Division of Pediatric Rheumatology, Department of Pediatrics, Duke University, Durham, NC, United States; ^3^Mayo Clinic, Rochester, MN, United States

**Keywords:** juvenile myositis/deratomyositis, outcomes, myositis, predictive model, biomarkers

## Abstract

Humans have an innate desire to observe and subsequently dissect an event into component pieces in an effort to better characterize the event. We then examine these pieces individually and in combinations using this information to determine the outcome of future similar events and the likelihood of their recurrence. Practically, this attempt to foretell an occurrence and predict its outcomes is evident in multiple disciplines ranging from meteorology to sociologic studies. In this manuscript we share the historical and present-day tools to predict course and outcome in juvenile idiopathic inflammatory myopathy including clinical features, testing, and biomarkers. Further we discuss considerations for building more complex predictive models of outcome especially in diseases such as juvenile idiopathic inflammatory myopathy where patients numbers are low. Many of the barriers to developing risk prediction models for juvenile idiopathic inflammatory myopathy outcomes have improved with many remaining challenges being addressed.

Humans have an innate desire to observe and subsequently dissect an event into component pieces in an effort to better characterize the event. We then examine these pieces individually and in combinations using this information to determine the outcome of future similar events and the likelihood of their recurrence. Practically, this attempt to foretell an occurrence and predict its outcomes is evident in multiple disciplines ranging from meteorology to sociologic studies. In medicine, the ancient Greek physicians, most notably Hippocrates and Asclepius, relied on examination and observation of patients to develop the art and science of diagnostic and prognostic medicine. The word prognosis (Greek: πρóγ*νωσις*) translates into “knowledge beforehand,” how an event is likely to conclude (1). The ability to make a prognosis is a tenet and legacy of Hippocratic medicine. The study of biological systems evolved from defining the essential character of an observation or occurrence, natural philosophy, to using empiric methods for descriptions of how they occurred, the scientific method. The evolution of scientific capabilities led to the ability to look at these systems on a microscopic and molecular level, to attempt to understand the larger entity by breaking it into the smallest component pieces, known as reductionism. Advances in medical research have paralleled the advances in biologic research. Currently, we are in an era of system level observation of cellular networks where high-throughput technologies allow further resolution of these systems and generate complex data. Multivariate statistical methods are integral to the analysis of these biologic networks and are vital tools in the effort to discover biomarkers that are predictive of disease activity, severity, and response to therapeutic interventions.

Predictive and prognostic models are statistical tools that predict a clinical outcome determined by at least 2 points of patient data and ideally more with novel statistical models which take into account change over time (2, 3). Adequate prediction of prognostic endpoints generally requires multiple prognostic factors (variables, predictors, or markers) (2). Therefore, the original dataset may contain numerous covariates including clinical and biological markers identified as potential predictors of disease characteristics (phenotype/severity, or outcomes). Development is a process that includes identification of a relevant pool of predictors, formulation of a statistical model that may employ techniques such as linear regression, logistic regression, or Bayesian models, among others. Once a model is developed, it undergoes internal and external validation (2, 3). In the context of patient care, the goal is to develop a model that predicts an accurate diagnosis based on entered data. Treatment tailored to that disease has the potential to limit morbidity associated with inappropriate therapy (4). This is important in a disease with numerous clinical phenotypes such as the Juvenile Idiopathic Inflammatory Myopathies (JIIMs). A second combination of data utilizing a different statistical model may predict disease course or prognosis. Prognostic models have advanced from basic decision rules (prediction rules) used at the bedside to aid diagnostic and clinical decision making into complex mathematical formulas developed based on large population databases (3, 5).

The JIIMs represent a rare heterogeneous group of systemic autoimmune vasculopathies characterized by variable involvement of the skin and muscle primarily, but with the potential to affect multiple organs. The most common JIIM is juvenile dermatomyositis (JDM). Other JIIMs include juvenile polymyositis (JPM), immune-mediated necrotizing myositis, and myositis associated with connective tissue disease (6). Prediction of course and prognosis in JIIM has been difficult. In 1983, Bowyer et al. stated, “It has been impossible to predict at the onset of juvenile dermatomyositis whether a child will have complete recovery…” (7) More recently, van Dijkhuizen reporting for the Juvenile Dermatomyositis Research Group (JDRG) stated, “It is currently impossible to predict the prognosis of patients with JDM” (4). Bowyer's group sought to identify factors present early in the course of disease that might determine significant morbidity looking at clinical and treatment variables. Van Dijkhuizen's group, 35 years later, reports employing a Bayesian model of disease activity utilizing four continuous outcome variables to stratify patients by disease activity and allow for more sign/symptom-specific treatment based on these variables. Logically, predictors of disease course and prognosis have historically paralleled development of capabilities to characterize and examine disease manifestations, first by description of signs and symptoms, and later by immunohistochemistry and molecular techniques (8–16).

The years just prior to and since the new millennium have seen development, validation, and revision of measures of disease activity, severity, and outcome in the JIIMs, particularly JDM. Organizations instrumental in development of these measures include the International Myositis Assessment and Clinical Studies Group (IMACS) and the pediatric Rheumatology International Trials Organization (PRINTO). These measures assess various domains of the JIIMs including global disease activity, muscle strength, physical function, and quality of life (Table 1) (14). Core set measures (CSMs) have been developed and validated for assessment of disease and treatment variables in JDM (16, 18, 19). CSMs are the minimum set of measures, in aggregate, that allow for adequate assessment of the disease within the various domains studied, and are required for implementation in all clinical and therapeutic trials (20, 21). In order for measures to be useful in clinical care and research, definitions of disease improvement, severity, and response to therapy need to be available. For example, response to therapy being defined as at least a 20% improvement in three of six CSMs with no more than one or two worsening (which cannot be muscle strength) had been established as preliminary response criteria employed by both PRINTO and IMACS (20).

**Table 1:** Disease related measures used in predicting disease severity and outcome (17).

**Domain**	**Measure**	**Grading**
Disease Activity—Includes extramuscular	Physician global activity Patient/parent global Disease Activity Score (DAS); developed for JDM Myositis Disease Assessment Tool (MDAAT) –Combined tool that includes the Myositis Disease Assessment VAS (MYOACT) and Myositis Intent to Treat Activities Index (MITAX)	Visual Analog Scale (VAS) or Likert scale Visual Analog Scale (VAS) or Likert scale 10 items scored dichotomously, 3 polychotomously; also DAS skin (range 0–9) and muscle (range 0–11) scores Combined tool: VAS for each organ (MYOACT) and polychotomous response (MITAX)
Overall Health Status	Child Health Questionnaire (CHQ)	Consists of 14 health concepts
Physical Function	Childhood Health Assessment Questionnaire (CHAQ) Childhood Myositis Assessment Scale (CMAS); physical function, muscle strength, and endurance in JIIM	Questionnaire measuring degree of difficulty performing activities of daily living (ADLs); VAS for pain assessment and overall well being Observational, performance-based grading
Muscle Strength	Manual Muscle Testing 8 (MMT8)	10-point scale; 8 muscle groups
Cutaneous Involvement	Cutaneous Assessment Tool (CAT)	Scoring based on lesion characteristics: 0–2 or 0–7 depending on item
Global Damage	Physician Global Damage Myositis Damage Index (MDI)	VAS or Likert scale 11 separate VAS ratings
Laboratory Assessment	Muscle enzymes (creatinine kinase, aldolase, LDH, AST, ALT)	

Predictive modeling utilizing clinical and laboratory data has been employed in JDM. Van Dijkhuizen's et al. utilized data from the UK Juvenile dermatomyositis cohort and biomarker study (JDCBS) in which data were analyzed using a Bayesian model to develop a model of disease activity (21). They identified signs and symptoms that associated with four outcome parameters. These parameters measured longitudinally included creatinine kinase (CK), childhood myositis assessment scale (CMAS), manual muscle testing 8 (MMT8), and physician global assessment (PGA). Among other associations, they discuss the association of periorbital rash with lower CMAS and higher CK values concluding this may support the opinion that ongoing skin disease reflects ongoing systemic disease activity (21). Deakin reporting for the Juvenile Dermatomyositis Research Group (JDRG) describes the use of marginal structural modeling (MSM) in determining the efficacy and safety of cyclophosphamide (CYC) treatment in severe JDM (22).

Marginal systems modeling (MSM), a statistical strategy utilizing multi-step estimation was employed by Lam et al. to adjust for baseline confounding bias to establish causal relationships using observational data between control and treatment groups in a cohort of JDM patients receiving IVIG (23). This study was significant in its application of bias-reduction methods to demonstrate the efficacy of IVIG in controlling JDM, most notably in corticosteroid-resistant patients. Deakin et al. also used MSM to determine the efficacy and safety of cyclophosphamide (CYC) in the treatment of severe JDM. The retrospective study consisted of 200 cases, 56 patients receiving, and 144 not receiving CYC (22). Descriptive analysis as well as MSM revealed improvements in three domains of disease activity (skin, muscle, and overall) in CYC-treated patients vs. those not treated. In the MSM analysis, the improvement was greatest at 12 months after the start of CYC in skin disease and global disease activity. Only minor adverse events were noted in three patients within 1 year of stopping CYC. In addition to efficacy and safety, the relative cost was lower and course of treatment was shorter using CYC as compared to biologic therapies.

Key factors for validation across universal cohorts is the ability to have comparable measures and outcomes. Work by CARRA, IMACS, PRINTO, cure JM, and JDRG all have worked to identify an international set of evaluations, measures for disease change and treatment protocols and place them into routine care of patients and not only in clinical trials (14, 16, 24–29).

In addition to clinical and laboratory measures, additional biomarkers are now being assessed as variables utilizing statistical methods to develop predictive and prognostic models.

## Historical Biomarkers

Our ability to predict outcomes in JIIM continues to be limited by our subjective clinical assessments and our minimal and insensitive laboratory data. Exploration into biomarkers has been ongoing and continues to include cytokines, dysregulated inflammatory markers, autoantibodies, and muscle tissue markers. Individual biomarkers hold promise to be informative. However, it is more likely that these measures in concert hold a stronger association than they do individually, as demonstrated with disease modeling.

Over the past few decades, the development of new technologies, specifically high-throughput systems, has allowed identification of markers such as individual proteins, RNA immune related elements, and autoantibodies as markers of disease. Many of these same markers have been studied longitudinally and related to disease activity markers.

## Transcriptional Analysis

**Figure 1:**
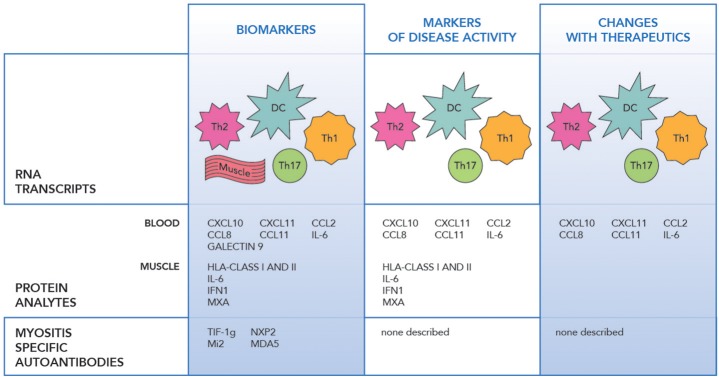
The figure illustrates the transcript, protein and antibody biomarkers in juvenile idiopathic myopathy. RNA Transcripts include DC dendritic cells (IFNα/β, OAS1, 2), Th1 (IL-1, CXCR3, FCGR1A), Th2 (IL-4, IL-13, and GATA3), Th17 (IL-6, IL17D, IL-17F, IL-21, IL-23A, IL-27, RORC/RORγt, and IRF4), Monocytes (CXCL9, CXCL10, CXCL11), Muscle (HLA class I and II, MX1 and MX2, MxA, SGF-15, RORc, STAT3, cytochrome C oxidase, and NADH dehydrogenase), IFNβ, IRF7 (24–34).

RNA profiling of peripheral blood, muscle and skin biopsies in JIIM demonstrated similar patterns of the activation of the innate immune system with type 1 interferon (IFN1) induced, and the adaptive immune system with IL-17 and IL-6 pathway involvement, along with Th1 and Th2 related transcripts dysregulation (Figure 1) (30–33). The earliest differentially expressed transcripts reported in JIIM muscle tissue included an increase in IFN1 and Human Leucocyte Antigen (HLA) class I and II. The upregulation of HLA class I is now used in the disease diagnosis (31, 33–37). Chemokines related to both monocyte and lymphocyte immune function, such as CXCL9 [Monokine induced by gamma interferon (MIG)], CXCL10 (also known as IP-10, interferon gamma-induced proteins), and CXCL11 [Interferon-inducible T-cell alpha chemoattractant (I-TAC)], along with Calcium Binding Proteins such as S100A10, TNFSF13B (BAFF), ISG15 an ubiquitin-like modifier, are all reported dysregulated in JIIM (30, 38–40). An early study demonstrated that a cluster of IFN-regulated transcripts are upregulated in most JIIM patients and consist of classical IFN-induced genes including STAT1, SOCS1, the myxovirus resistance genes MX1 and MX2, the oligoadenylate synthetase transcripts OAS1, OAS2, and OAS3, Fcγ receptor FCGR1A, the interleukin 1 receptor antagonist (IL1RN), the pro-apoptotic TNFSF1 0/TRAIL, and another inflammatory chemokine CXCL10/IP-10 (30, 37).

Non-immune genes are also differentially expressed and include transcripts related to the oxidative pathways and mitochondrial function such as cytochrome C oxidase and NADH dehydrogenase (30, 33).

Not only have these transcripts been reported as dysregulated in JIIM, but they have also been reported to correlate with disease activity measures. Many individual transcripts were statistically significant. However, as with other disorders such as Systemic Lupus and JIIM, Baechler et al found combinations of interferon related transcripts specifically built as interferon scores appeared to be more reliable and withstand longitudinal disease variability including treatment (30, 41, 42). Further refinement of the IFN gene signature in JIIM was determined using the expression levels of 3 IFN-regulated genes (IFIT1, G1P2, and IRF7) using quantitative real-time reverse transcription-polymerase chain reaction and normalization to obtain an IFN score (41). This was later verified and used in adult myositis (40). This also led to the development of an IFN protein score with proteins found to be associated with disease activity. (MCP-2, CXCL10, and CXCL11) (25, 36).

## Protein Markers

Just as microarray technologies have allowed identification of new biomarkers, advances in proteomics have allowed identification of protein changes with disease states and interventions. A variety of proteins, including levels of IFN regulated proteins included anti-viral proteins, humoral and adaptive immune proteins, and chemokines are seen in the peripheral blood, in specific cell subsets and in the muscle tissue in JIIM. Specific analytes include IFN alpha and beta, IL-6, IL-17, chemokines including MCP-1/CCL2, MCP-2/CCL8, IP-10/CXCL10, I-TAC/CXCL11, IFNγ, Galectin 9, IL-1Ra, GM-CSF, and Eotaxin (Figure 1) (41, 43–46). Markers including those related to IFN1 upregulation along with IL-6, CCL11, MCP-1, CXCL11, and CXCL10 appear to hold the strongest correlation with disease activity in JIIM (37, 41, 43, 45, 47, 48).

Many of these proinflammatory cytokines induce or enhance the metabolic effects on muscle tissue, especially during the regeneration process (49). Cytokines such as type-1 interferon, IL-1, and TNFa are upregulated in myocytes along with the increased expression of HLA-class I. In addition, with myocyte regeneration, seen in IIM, there also is upregulation of HLA-class I along with type-1 IFN and IL-6, which could lead to further inflammation (44, 50–53).

Tissue-specific markers, such as those related to atrophic myofibers, include the ISG15-conjugation pathway proteins such as MxA, which are upregulated in active disease. Several IFN-regulated chemokines showed significant positive correlations with muscle enzymes, including MCP-1, MCP-2, and CXCL10. Baechler (30) and Bilgic (41) demonstrated that JIIM with the highest degree of disease activity had elevation of IFN- regulated proteins CXCL10, MCP-1, and MCP-2. Similar to RNA transcripts, scores using multiple transcripts and multiple protein analytes combined together created a stronger association with disease activity measures (41). The set of JIIM markers indicate the intricate matrix and interconnectedness of the innate, humoral and adaptive immune systems in autoimmunity.

## Autoantibodies

Identification of a myositis specific autoantibody (MSAs) is highly suggestive of an inflammatory myositis and more recently recognized in JIIM. This has led to autoantibody relationships with clinical disease phenotypes, and possibly antibody levels themselves fluctuate with disease activity. Those more commonly seen in JIIM include anti-TIF 1(Transcriptional intermediary factor 1), NXP2 (Nuclear Matric protein 2), MDA5 (Melanoma differentiation-associated gene 5), Mi2 (Nucleosome-remodeling deacetylase complex), and less commonly anti-SAE, ASA (synthetase), SRP (Signal recognition particle and HMGCR (3-hydroy-3-methlglutaryl coenzyme A reductase (54–56).

Myositis-specific antibodies have an increasing utility as both diagnostic and prognostic biomarkers with multiple publications suggesting clinical features related to antibody specificity (54–56) (Figure 1). Not only are MSAs markers of the clinical phenotype, but they have also been investigated as disease activity markers. Myositis-associated antibody levels for anti-Jo-1 TIF1-γ, SRP, and -Mi-2 were investigated after B cell depletion in adult and pediatric DM and adult PM and correlated with disease activity. Anti-Jo-1 serum levels correlated with clinical improvement specifically MMT and muscle enzymes (*p* = 0.007) (17). In DM patients who had anti-CADM-140/MDA5 autoantibodies and rapidly progressive interstitial lung disease, the mean titer of anti-CADM-140/MDA5 of anti-CADM-140/MDA5 significantly decreased in the responder group compared to non-responders (*P* = 0.033) (57).

In the future, predicting disease response to treatment as well as disease- and treatment-related outcomes will require classification of myositis patients in more homologous groups than the traditional PM and DM subtypes.

### Predicting Disease Outcomes in JIIM

While risk prediction models have recently become commonplace in the medical literature, there have been few attempts to develop risk prediction models in patients with JIIM. This is likely due to the rarity of the disease, as a minimum sample size of 200 patients is preferred for risk prediction models. In addition, the heterogeneity of disease activity and therapeutic strategies in patients with JIIM has made risk prediction difficult in this patient population. For example, a small observational study of 39 patients with JIIM, among whom six achieved clinical remission, found that female sex, negative Gower's sign and photosensitivity were associated with achievement of complete remission, but the small sample size precluded development of a multivariable model that could be used for prediction purposes (58).

Cooperative efforts including registries and biobanks are now making this task more feasible (59). Challa et al. recently published a model to predict changes in disease activity among children with JIIM using the Childhood Arthritis and Rheumatology Research Alliance (CARRA) Legacy Registry (60). They found that anti-nuclear antibody (ANA) positivity and use of hydroxychloroquine predicted improvement in patient/parent global health score over 6 months, and ANA positivity along with V/shawl sign predicted improvement in patient pain.

The Rituximab in Myositis (RIM) trial also provided opportunities to develop risk prediction models in patients with refractory disease. Aggarwal et al. (45) found that the presence of anti-synthetase and anti-Mi-2 autoantibodies and lower disease damage strongly predicted clinical improvement in these patients, and that the juvenile patients had better prognosis than the adults (61). Reed et al. found that biomarker signatures involving type-1 interferon regulated and other proinflammatory chemokines and cytokines in conjunction with autoantibodies predicted response to rituximab in patients with refractory myositis (45). Furthermore, Olazagasti et al. found that adding gene expression, cytokine and chemokine data to clinical and standard laboratory assessments improved prediction of response to rituximab in patients with JIIM (62).

### Practical Considerations for Building Predictive Models in JIIM

In addition to the importance of a sufficient sample size, there are several other important considerations for building risk prediction models. First, the study design and population of interest should be considered. As mentioned above, registries are making risk prediction in JIIM more feasible. While registries are observational studies, which may suffer from confounding, they can be used to predict outcomes from baseline characteristics. However, confounding by indication is common, as patients with more severe disease often require more therapies and interventions. This confounding can make it difficult to assess the impact of treatment on the outcome of interest. Alternatives to address confounding my indication are causal inference analysis methods (e.g., MSM) or randomized clinical trials. By randomizing patients to receive a treatment, confounding by indication is eliminated. However, clinical trials also have limitations because they often have strict inclusion criteria. Thus, patients enrolled in clinical trials may not represent the population of interest, as some of the patients for whom the risk prediction model could be useful were excluded from the trial.

Risk factor selection is another important consideration when developing a risk prediction model. The existence and assessment of relevant disease activity measures and biomarkers, such as those mentioned previously in this review, is critical. Without reliable disease activity measures, it is not possible to build models that will predict disease-related outcomes. In addition, causality is important, because when factors included in a risk prediction model improve, it is assumed that the risk of the outcome will be reduced. However, proving causality is difficult and requires causal inference analysis methods or a randomized clinical trial. Finally, the practicality and cost of each potential risk factor needs to be considered. Measurements that are financially costly or those that require a great deal of effort to assess, such as cumulative measures, provide challenges for implementing a risk prediction model in clinical practice.

The choice of model is also an important consideration. Historically, logistic regression models for cross-sectional data and Cox regression models for longitudinal time-to-event data were commonly used for risk prediction. These models had the advantage of providing an easily understandable equation for predicting the risk of an outcome. However, they were often overly simplistic and only considered linear effects of the continuous risk factors. More recent advances in statistical methods, which evolved from computer science methodology, including machine-learning techniques, have demonstrated improved performance for risk prediction models resulting from complex algorithms that do not provide simple risk calculation formulas. Computing advances have made it much easier to implement these complex algorithms into clinical practice using web-based tools, so simple formulas are no longer required and has opened the possibility of novel algorithms to apply to complex biomedical datasets.

Finally, once the risk prediction model has been developed, it is important to objectively assess its performance. Model performance assessment should include both discrimination and calibration. Discrimination is the ability to correctly rank patients from low to high risk. Calibration is the ability to accurately predict the absolute risk level. There is a wealth of literature on how to assess model performance for different types of models, which is beyond the scope of this review.

Risk prediction models should be validated prior to use in clinical decision-making. External validation in a separate dataset is preferred. However, internal validation based on subdividing the study patients into training and test dataset is also common. Szodoray et al. randomly subdivided their patients into 50 cases and 50 controls for training and 29 cases and 20 controls for testing (44). Analyzing multiplex cytokine assays using principal components, hierarchical clustering and discriminant function analyses, they identified unique immune profiles that seem to perpetuate autoimmune processed in patients with JIIM and may be able to identify disease subsets. Many of the modern modeling techniques use cross-validation methods to avoid over-optimism or over-fitting of a risk prediction model. None of the previously mentioned risk prediction models for JIIM have been validated due to study sample sizes that were too small to subdivide and a lack of available external data sources for validation.

In conclusion, while many of the barriers to developing risk prediction models for JIIM outcomes have improved, there are still many remaining challenges. The availability of registries to provide larger sample sizes and newer biomarkers that can predict outcomes has reduced the challenges, but careful planning will still be required to navigate these challenges and ultimately develop useful risk prediction models for patients with JIIM. However, there continues to be a major need to compare across data sets and cohorts that require continued standardization and validation across cohorts and centers.

## Author Contributions

All authors listed have made a substantial, direct and intellectual contribution to the work, and approved it for publication.

### Conflict of Interest Statement

The authors declare that the research was conducted in the absence of any commercial or financial relationships that could be construed as a potential conflict of interest.
